# Partial Immersion Using Body Bags for Exertional Hyperthermia: A Randomized Crossover Study

**DOI:** 10.5811/westjem.62965

**Published:** 2026-09-05

**Authors:** Diane Zashin, Xiujing Zhao, Luzita Vela, Elizabeth Parke, Ramon Ylanan, Brendon P. McDermott

**Affiliations:** * Wellness Workdays, Hingham, Massachusetts; † University of Arkansas, Heat and Hydration Optimization (H2O) Lab, Fayetteville, Arkansas; ‡ University of Arkansas, Department of Health, Human Performance and Recreation, Fayetteville, Arkansas; § University of Arkansas for Medical Sciences, Department of Orthopaedics and Sports Medicine, Fayetteville, Arkansas

## Abstract

**Introduction:**

Recent recommendations suggest emergency medical personnel should consider rapid whole-body cooling for heat stroke patients. Cold-water immersion maximizes cooling rate and subsequent outcomes following heat stroke. Body bags filled with ice and water may provide portable, accessible partial cold-water immersion for this setting. The purpose of this study was to determine whole-body cooling rates when using body bags to facilitate partial cold-water immersion to treat exertional hyperthermia.

**Methods:**

A total of 9 healthy participants (8 male; mean [SD] age, 24 [4] years; height, 175 [7] cm; body mass, 83.6 [21.6] kg) completed our randomized-crossover field study. Following hydration verification, participants completed a self-paced 400 warm-up run, 1,609 m run, and 10-m sprints until rectal temperature (T-*_rec_*) reached 39.2 °C or volitional exhaustion. Following exercise, participants were cooled for a maximum of 30 minutes lying supine in the shade (control; CON) or in body bags filled with 20 gallons of ice water (5.2 [1.6] °C). Following cooling, participants sat upright for a 15-minute recovery. T-*_rec_* and heart rate (HR) were taken throughout exercise, every minute of cooling, and every 5 minutes of recovery. Participants completed both trials separated by 1 week in random order.

**Results:**

Wet bulb globe temperature between trials was not significantly different (grand mean, 28.43 [1.03] °C; *P =* .73). There was no difference in participant maximum T-*_rec_* (body bag, 38.98 [.23] °C; CON, 38.93 [.39] °C; *P* = .45); HR (body bag, 183 [14] beats per minute [bpm]; CON, 189 [13] bpm; *P* = .21); rating of perceived exertion (*P* >.99); thirst (*P* = .62); or thermal sensation (*P =* .73) during exercise. Fluid consumed (*P* = .40), body mass change (*P* = .90), 400 m run times (*P* = .19) and 1,609 m run times (*P* = .17) were no different between trials. Whole-body cooling rate for body bag was .102 (.04) °C/min, whereas CON produced a cooling rate of .040 (.04) °C/min (95% CI, .030–.095; *P* = .002). Total cooling time was significantly faster (13.2 (5.8) min) with BB compared to CON (27.6 (4.2) min; 95% CI, 9.2–19.5; *P* = .002).

**Conclusion:**

In clinical settings where full-body cold-water immersion is not feasible, or during emergency transport, partial immersion via body bags offers acceptable cooling rates for hyperthermic patients. Emergency medical personnel could apply these data for support in updating heat stroke protocols to include partial immersion via body bag during transport, or immediately upon emergency department arrival.

## INTRODUCTION

Heat stroke represents the most serious heat illness, with potentially fatal consequences unless rapid recognition and treatment are initiated soon after onset.^1^–^4^ Clinical recommendations for the management of exertional heat stroke demonstrate overwhelming success when early recognition and effective treatment are initiated.^5^ Evidence-based recognition and management could improve outcomes within military and emergency department (ED) settings. The Army and Marine Corps have higher incidences of exertional heat stroke with .67 per 1,000 person-years and .72 per 1,000 person-years, respectively.^6^ However, the feasibility of having cold-water immersion tubs available for all training is limited.^6^ In EDs, the recognition and treatment of either classic or exertional heat stroke also present challenges.^7^,^8^ In one analysis, 5.4% of those admitted to the hospital for heat illness were diagnosed with heat stroke.^9^ Those who were diagnosed had an admittance rate of 62.0%.^8^ In some areas, heat stroke cases represent 28.3% of all admissions and 77% of all deaths for heat illness cases.^8^ These numbers are the result of medical personnel on-site not following a “cool first, transport second” protocol, as well as a lack of resources for rapid cooling upon ED arrival.^9^,^10^

Military and ED settings include specific barriers for compliance with established, recommended practices for exertional heat stroke.^6^ Following rapid recognition in the field, effective treatment should be applied to avoid negative outcomes or sequelae.^10^–^12^ Data strongly suggest 100% fatality avoidance with whole-body cold-water immersion.^5^,^10^ In emergent situations, the standard of care is to confirm diagnosis (core temperature > 40.5 °C with central nervous system dysfunction) and immerse the patient. Recent prehospital care recommendations echo these guidelines.^1^,^11^,^12^ Treatment for classic heat stroke, however, lacks consistent supportive data.^8^,^12^ Recent cases support aggressive cooling for classic heat stroke patients as well.^13^–^15^ In either case, if lifesaving treatment involves reducing hyperthermia as quickly as possible, data support the use of cold-water immersion.^5^,^13^ Further, to facilitate effective cooling, using the coldest water possible and including water circulation should be included. Recent cases demonstrate successful outcomes for patients with exertional or classic heat stroke treated with cold-water immersion.^16^,^17^

Common issues reported include the logistics of transporting a large rigid tub in various environments and having access to water and ice volumes necessary for effective cold-water immersion. Both venues present a unique opportunity based on likely availability of body bags. Since cold-water immersion in a large rigid tub— the current gold standard for whole-body cooling^17^—is not always feasible at the site of heat stroke or during transport, body bags present a conduit to perform partial immersion.^4^,^13^ According to McDermott et al,^17^ ideal cooling rates for patients with exertional heat stroke are ≥ .155 °C/min, acceptable cooling rates are those between .078 °C/min and .154 °C/min, and unacceptable rates are those < .078 °C/min. Data are sparse on cooling rates for hyperthermic individuals in body bags.

**Population Health Research Capsule**

What do we already know about this issue?

*Cold-water immersion provides the fastest cooling rates for hyperthermic individuals and leads to superior patient outcomes*.

What was the research question?

*We sought to compare cooling rates for partial immersion in a body bag against lying in the shade for hyperthermic individuals*.

What was the major finding of the study?

*Cooling rate for body bags was .102 (.04) °C/min, whereas lying in the shade was .040 (.04) °C/min (95% .030–.095; P = .002)*.

How does this improve population health?

*Using a body bag to facilitate whole-body cooling via partial immersion is an option for treating hyperthermic patients in remote settings or during transport*.

Studies have been sponsored by a manufacturer of the Polar Life Pod, a body-bag–like device including a pillow and straps (Polar Products, Inc, Stow, OH). Miller and Beck^18^ reported efficient cooling using the device with water oscillations (between .10 and .17 °C/min) with varying water volumes. Further, Miller and Amaria^19^ reported ideal cooling with constant oscillations using different water temperatures. Nye et al,^20^ however, in the only study using the Polar Life Pod without manufacturer funding, found inadequate cooling rates (.04 °C/min). The device may not be a feasible alternative in emergency medical services situations given the cost (> $400); however, traditional body bags, already available in emergency settings, offer a more affordable option.

There are case report data supporting cooling with body bags.^4^,^10^,^13^–^15^ However, without documented support with consistent cooling rates, clinicians should use caution when applying body-bag partial immersion. Offering this alternative could demonstrate an effective cooling modality and improve compliance and patient outcomes. Body bags filled with ice and water, which can fully encase a patient, offer an alternative method of cooling while increasing portability and accessibility for other healthcare professionals (emergency physicians, nurses, etc) and tactical settings. An alternative cooling modality that produces acceptable or ideal cooling rates could be used to treat heat stroke in settings where access to cold-water immersion tubs or a commercial cooling device is limited, or not feasible.

The purpose of this study was to determine whether partial immersion cooling via body bags offers cooling rates that can be deemed appropriate for cooling hyperthermic patients. Our hypothesis was that partial immersion using a body bag would produce acceptable or ideal cooling rates, making them appropriate to recommend for partial immersion as an alternative treatment when cold-water immersion is not feasible.

## METHODS

We used a randomized, crossover research design in the field to test our hypothesis. The University of Arkansas Institutional Review Board (IRB) approved the study. A total of 13 volunteers were recruited from the University of Arkansas and surrounding area. Each participant completed a medical questionnaire screening to determine fitness and health levels as well as assuring inclusion criteria were met. One participant did not meet inclusion criteria (taking medication known to affect thermoregulation) and was excluded from our study. Participants were included if they self-reported physical activity for at least 30 minutes three times a week and were under the age of 40 years. Participants were excluded if they had suffered from heat illness or exertional heat stroke in the prior three years, were taking medication known to alter fluid balance and/or thermoregulation, were sick at the time of testing, or had suffered a recent orthopedic injury or had exercise restrictions due to an injury. Once clearance was given, participants were randomly assigned to participate in two trial conditions, with the order of treatment for trials randomized using an online random number generator. Researchers and participants were unaware of the trial order for each participant until they arrived and signed their consent form. Based on previous data on cooling rates within our lab, and to garner an estimated power of .80 with significance set to < .05, we calculated that a sample size of eight was required.

Participants were randomly assigned to a control (CON) or body-bag cooling trial, with allocation concealed; trials occurred one week apart. We chose not to include a trial of full-body cold-water immersion because those cooling rates have been fully established.^17^ Further, because cooling rates from exercise hyperthermia match those from case series data, the established cooling rates following exercise hyperthermia are assumed similar to actual heat illness scenarios.^4^,^16^,^17^ On the day of the trials, participants reported to the recreational fields at the University of Arkansas at their allocated time. Each subject read through and signed the IRB-approved consent form, and participants could question the research team. Following consent, each participant submitted 24-hour food and fluid logs, which they were instructed to complete for the day leading up to trials to ensure similar fueling and hydration status between trials. They also affirmed that they had not consumed alcohol within 24 hours or participated in exercise within 12 hours of the study trial.

Wet bulb globe temperature was taken every 15 minutes during data collection (Kestrel 4500, Boothwyn, PA). Following consent and paperwork, body mass was measured with a ground scale (Health-o-meter model 349KLX, Buford, GA), height was obtained using a stadiometer, and pretrial urine specific gravity (USG, Master-SUR, Atago Co Ltd, Tokyo, Japan) was obtained to confirm euhydration, which was established as < 1.020. Participants were then instrumented by self-inserting a rectal thermistor (RET-1, Physitemp, NJ) 15 cm past the anal sphincter. To confirm proper instrumentation, a preliminary reading was obtained. If there were invalid readings (< 36 °C or > 38 °C), participants were instructed to further insert the thermistor, or the thermistor was exchanged. Researchers then fit and calibrated participants with a GPS and heart rate (HR) watch (Ironman Triathlon, TIMEX, CT). Both the rectal thermistors and watches were number labeled and matched between trials. Participants were instructed to wear comfortable clothing for exercise and cooling (with potential immersion). Clothing during exercise and cooling was matched for each participant between trials. Participants were considered heat acclimatized due to data collection occurring in September in a mid-southern state.

Our exercise protocol was based on previous work demonstrating safe rectal temperature (T-*_rec_*) increases in a relatively short time period (18–20 minutes). Participants began their exercise trial with a 400 m self-paced warm-up. Following the 400 m warm-up, they were instructed to complete a 1,609 m run (1 mile) at their own pace around the fields. Following the warm-up, researchers collected (T-*_rec_*), HR, and perceptual measures. Perceptual measures included rating of perceived exertion, thirst, and thermal sensation. Participants had 5 minutes to walk around, rest, stretch, and drink water at their discretion. They were also informed that they were not permitted additional fluids for the remainder of the trial. Water consumed was measured in mL following the 5-minute rest and matched between trials. Then, participants began their 50-yard out-and-back sprints for a total of 100 yard sprints. A set was made up of 5 sprints separated by 30 seconds rest between each out-and-back. Once a set of sprints was completed, T-*_rec_*_,_ HR, and perceptual measures were recorded during a 3-minute rest period. Participants repeated sets of sprints until either (1) T-*_rec_* of 39.2 °C or (2) unwillingness to continue due to exhaustion or signs/symptoms of heat illness presented. When either of those criteria were met, the 5-minute transition to cooling commenced. Transition included a postexercise body mass measure, which was used to calculate sweat rate during exercise.

Cooling was completed in the shade for both conditions, either immersed in a postmortem bag (Cardinal Health Inc, Dublin, OH) with cold water or sitting in the shade. Blinding of participants, researchers and data recorders was not possible during cooling. During the transition, two 10-gallon coolers were filled with one 5-lb bag of ice and water. The body bag was prepped in the grass field under a shaded tent with four seatbelts (Xuesong, China) wrapped around the bag and a half pool-noodle at the participant’s head to keep it propped throughout cooling (Figure 1).

Following the 5-minute transition period, perceptual and physiological measurements were taken before cooling began. Body-bag cooling occurred while participants were supine in the bag with 20 gallons of ice water poured on them and the bag zipped and seatbelts closed and cinched around the participant’s body.

Four to six researchers surrounded the participant while continuously oscillating the water in the bag. Throughout cooling, T-*_rec_* and HR were measured every minute with perceptual measurements taken following treatment. The. CON cooling occurred with the participant lying in the shade on a tarp. The cooling portion of the study was complete when a T-*_rec_* of 37.9 °C or 30 minutes of cooling was reached, regardless of trial. After cooling ceased, participants sat upright for 15 minutes to assure recovery from cooling.

Following recovery, the trial concluded and investigators removed the monitoring equipment. Participants returned their gear and received hydration and snacks prior to departure. Trials were separated by seven days, and participants who completed both trials were compensated for their time.

Sweat rate was calculated according to recommended assessment.^21^ Data were analyzed using SPSS for Mac (version 24.0; IBM Corp, Armonk, NY). Data for variables with two time points including wet bulb globe temperature, hydration status, and sweat rate were compared using paired samples *t*-tests. All data for variables with multiple time points were compared using repeated measures analysis of variance. When sphericity was violated, Greenhouse-Geisser corrections were used for interpretation. When a significant time point or interaction existed, post hoc analyses were conducted using Bonferroni correction. Data are presented using mean (SD), and alpha was set a priori as *P* < .05.

## RESULTS

Two participants did not begin either trial due to outside conflicts preventing participation. One participant suffered a musculoskeletal injury outside data collection following the first trial and could not complete the second trial. Any data gathered from these individuals were not used in the final analysis. Data for 9 participants (8 male; 24 [4] years, 175 [7] cm, 83.6 [21.6] kg); body surface area, 1.99 [.11] m^2^) were included.

Body mass (83.59 [21.61] kg; *P* = .90) and wet bulb globe temperature (28.43 [1.03] °C; *P* = .73) were similar between trials. Mile (1,609 m) times between trials (T1: 515.11 (132.21) seconds vs T2: 491.56 (102.61) seconds) were not significantly different (P = .40). There was no difference in rating of perceived exertion (15.39 [2.72]; *P* > .99), thirst sensation (6.12 [2.11]; *P*= .62), or thermal sensation (6.32 [.873]; *P* = .73) throughout exercise. Physiological measurements of HR (body bag, 183 [14] bpm; CON, 189 [13] bpm; *P* = .21) and maximum T-*_rec_* (body bag, 38.98 [.23] °C; CON, 38.93 [.39] °C; *P* = .45) showed no significant difference between trials.

Cooling rate via body bag was .102 (.04) °C/min whereas CON demonstrated a rate of .04 (.04) °C/min (95% CI, .03–.095; *P* = .002 (Figure 2). Body surface area was not significantly correlated with body bag (*r* = .20; *P* = .60) or CON cooling rate (*r* = −.082; *P* = .84). Water temperature just prior to pouring into the body bag with participants was 5.2 (1.6) °C. During cooling, thermal sensation was significantly lower with body bag treatment than with CON (*P*<.001). Total cooling time for CON was 27.6 (4.2) minutes and 13.2 (5.8) minutes with a body bag (95% CI, 9.2–19.5; *P* = .002). Data for cooling times for each participant between trials are found in Figure 3.

## DISCUSSION

The purpose of this study was to examine the use of partial immersion using a body bag to facilitate whole-body cooling following exertional hyperthermia. Affirming our hypothesis, the body bag method produced faster cooling rates than lying in the shade. Importantly, the body bag produced an “acceptable” (between .078 °C/min and .154 °C/min) cooling rate of .102 (.04) °C/min, making it a viable treatment option that could be included in prehospital care for heat stroke.^1^–^4^,^11^,^17^ It is important to recognize that a body bag did not achieve ideal cooling rates often associated with cold-water immersion. Emergency medical personnel should continue using whole-body cooling modalities demonstrating 100% survival rate if presented with heat stroke, when feasible.^1^–^4^

Body-bag cooling demonstrates successful results due to thermodynamic principles; similar to tarp-assisted cooling with oscillations (TACO).^22^,^23^ The key to success for TACO cooling includes immersing the patient in as much cold water as possible and facilitating oscillations. This fosters convective cooling, which accentuates heat transfer from the body. The body-bag method facilitates both principles, making this modality successful in whole-body cooling. Also, water oscillations are typically facilitated during patient transport (driving, turning) to the ED, which suggests that a body bag is an effective prehospital modality. Further, our results are similar to cooling induced using the Polar Life Pod following exertional hyperthermia.^19^,^20^ It should be noted that these previous studies used approximately 40 gallons of ice water on the participant to accelerate cooling, which may be a prohibitive amount of water for military and ED settings. Based on thermodynamics, body-bag cooling rates should approximate those documented with the Polar Life Pod given consistent clinical application requiring only 20 gallons of ice water.^17^ Further, body-bag cooling produces far superior cooling to ice sheet cooling,^7^,^24^ which persists in some military settings. Therefore, a body bag represents an effective and more affordable alternative for partial immersion whole-body cooling.

Ethically, participants were not allowed to reach the temperature threshold required for heat stroke (> 40.5 °C). Pathophysiology shows that while exertional heat stroke overwhelms the thermoregulatory and cardiovascular systems, they do not shut down.^3^,^8^,^12^ This is similar with exertional hyperthermia, as the thermoregulatory system is overwhelmed but does not fail.^3^ This principle allows for a study to simulate comparable heat stress to those who suffer heat stroke. Previous data justify extrapolating exertional hyperthermia cooling rates to those expected for exertional heat stroke.^5^ Previous literature presents similar cooling rates between exertional heat stroke and exertional hyperthermia using the same modality, making it acceptable to infer expected cooling rates for diagnosed heat stroke patients based on data from hyperthermic participants.^4^,^5^,^17^,^18^

There are clear benefits for the use of a body bag in certain settings compared to cold-water immersion using a large, rigid tub. Body bags can be folded and stored in confined spaces, offering a portable cooling alternative ideal for prehospital and athletic training settings. They are also readily available and accessible in emergency response situations. Further, internet searches on global shopping websites produce a wealth of options. Based on preliminary pilot testing, two 38-L coolers of ice water were poured over participants to maximize immersion depth while minimizing fluid leakage through the zippered seams of the body bags. Body bags for partial immersion could be used in areas of remote endurance events and in military training. These settings could use partial cold-water immersion in a portable and feasible manner to treat hyperthermic patients. Smaller amounts of water may also be effective^19^ but were not tested in our study.

We found it ideal to have approximately five people involved in cooling a single patient with a body bag. One person should remain at the head, keeping the head elevated, with two people on each side of the patient oscillating the water. Other clinician or volunteer numbers could also work as well. It should also be noted that for this cooling medium, an individual needs to be enclosed within the bag and secured with straps, which can be difficult if they are demonstrating central nervous system dysfunction.^1^ It is crucial that there is proper water immersion and oscillation, and if either cannot be achieved due to symptoms, another more effective cooling modality should be considered.

## LIMITATIONS

There are a few limitations associated with our study. An important limitation was the importance of the thermodynamic principle of convection and its relation to body surface area. Cooling modalities that have a larger body. surface area covered with ice water increase heat transfer and cooling rate.^27^,^28^ Given that we standardized the amount of water used in a body bag for each participant, participants with greater body surface area had less depth and partial immersion of ice water. To achieve convection, physical labor was required to move that participant within the body bag.

Participants with a larger body surface area required greater physical effort to oscillate due to the increased total mass of the bag. This mechanical limitation may explain the negative correlation observed between body mass and whole-body cooling rates. Studies show that the ratio of fat mass to fat-free mass may limit cooling rate.^27^ This could warrant research on a specific metric of water immersion necessary to achieve effective cooling rates, rather than a standardized amount of water. We selected an ice water volume that optimizes clinical feasibility across a wide range of athletic, military, and emergency medical settings. An additional limitation was minor body-bag malfunctions. Water leaking from the bags and pooling at the feet likely limited the amount of cold water available for convective and conductive heat transfer. In the future, research should be conducted on a type of body bag and cinching material that produces the least amount of water leakage while maintaining maximal immersion around the thorax.

Given our limited sample size of 9 participants who were otherwise healthy, the study may not reflect the effect on actual patients, although cooling rates compared between patients with exertional heart stroke^5^ and hyperthermic, healthy participants^18^ typically align. Finally, a clinical limitation of the body bag used in this study is its black coloration, which is typically associated with deceased individuals and could potentially induce psychological distress or patient noncompliance. However, our findings suggest that medical equipment manufacturers could produce structurally identical. Pouches in neutral colors explicitly for hyperthermia management.

## CONCLUSION

Research continues to evaluate the outcomes of exertional heart stroke and proper treatment measures. In the ED, during emergency transport, or where cold-water immersion tubs are not readily available, body bags filled with ice water can serve as a medium to adequate cool a heat stroke victim. Based on our data, in settings where full-body immersion is not feasible, partial immersion cooling via a body bag offers an effective alternative for managing hyperthermia. This could be considered for emergency action plans or emergency protocol when heat stroke is an inherent risk.

## Figures and Tables

**Figure 1:**
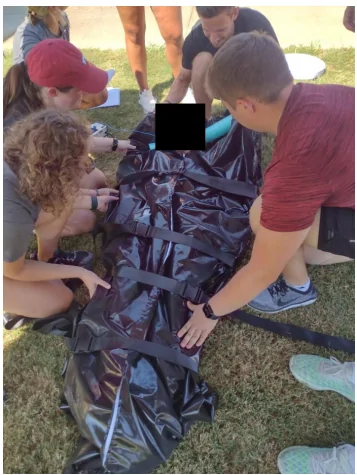
Body bag cooling set-up to treat a participant in a field study to determine whole-body cooling rates when using body bags to treat exertional hyperthermia.

**Figure 2:**
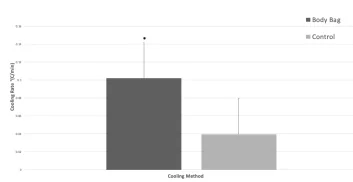
Cooling rate of body bag compared to control in study to determine whole-body cooling rates when using body bags to treat exertional hyperthermia.

**Figure 3:**
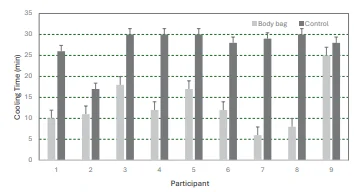
Cooling time in a body bag compared to control, based on a crossover study comparing whole-body cooling between partial immersion with body bags and lying in the shade as the control condition.
